# Validity and Reliability of the Turkish Version of the Healthy Aging Perception Scale in Older People with Chronic Diseases

**DOI:** 10.3390/bs15081048

**Published:** 2025-08-01

**Authors:** Nihan Türkoğlu, Nur Özlem Kılınç, Esin Kavuran

**Affiliations:** 1 Department of Public Health Nursing, Nursing Faculty, Ataturk University, Erzurum 25240, Turkey; 2 Department of Public Health Nursing, Fırat University, Elazığ 23119, Turkey; nokilinc@firat.edu.tr; 3 Faculty of Nursing, Nursing Department, Ataturk University, Erzurum 25240, Turkey; esin.kavuran@atauni.edu.tr

**Keywords:** healthy, chronic diseases, older people, validity, reliability, perception

## Abstract

In order to promote healthy aging, it is important to know older people’s perceptions of healthy aging. The aim of this study is to conduct a Turkish validity and reliability study of the Healthy Aging Perception Scale for Older People with Chronic Diseases. This study was conducted between November and December 2023 with older adult people living in a region in eastern Turkey. A total of 210 older people were included in the sample for the exploratory factor analysis and 214 for the confirmatory factor analysis. A Sociodemographic Information Form and Healthy Aging Perception Scale was used to collect the data of the study. The collected data were analyzed using SPSS 27.0 and AMOS 22.0. Confirmatory factor analysis for the 18-item scale, a four-factor structure with an eigenvalue exceeding 1, was obtained, and it was determined that the factor loadings ranged between 0.834 and 0.637 and the total variance explained was 64.619%. The confirmatory factor analysis of the goodness of fit indices was found to be at an acceptable level. CMIN/DF = 2.834, RMSEA = 0.05, CFI = 0.929, TLI = 0.910, NFI = 0.920, and GFI = 0.901. Cronbach’s alpha of the scale was determined to be 0.826, and the test–retest reliability coefficient was determined to be 0.822. Factor analysis showed a better model fit, and it was determined that the Healthy Aging Perception Scale is a valid and reliable measurement tool in determining the perceptions of the healthy aging of older people with chronic diseases.

## 1. Introduction

Aging is an unavoidable life process characterized by complex aspects that lead to a gradual decline in mental and physical abilities over time (Minarti & Kastubi, 2019; Sönmez & Çevik, 2021; WHO, 2024; Yerli, 2017). Recent advancements in medical technology and the implementation of early diagnosis and treatment have resulted in a decrease in disease-related deaths. Consequently, life expectancy has increased, leading to a growth in the elderly population (Kızılkaya & Atuğ, 2023). 

The rapid increase in the aging population is most pronounced in developing countries. Turkey, as one of these developing nations, is experiencing a swift rise in its elderly population, which presents numerous challenges that need to be addressed (Şeker & Kurt, 2018). In 2015, the World Health Organization (WHO) introduced a policy focused on healthy aging (WHO, 2015). Following this, the United Nations Digital Library declared the Decade for Healthy Aging (2021–2030) in 2021, considering the impact of the COVID-19 pandemic on older adults. According to the United Nations Digital Library, the percentage of people aged 65 and over rose from 6% in 1990 to 11% in 2019. The WHO Global Strategy and Action Plan on Aging and Health projects that the proportion of individuals aged 60 and over will double to 22% between 2000 and 2050 (United Nations Digital Library, 2019; WHO, 2015). 

As global population aging becomes a significant socio-economic challenge, policymakers are emphasizing the promotion of healthy and independent living among older adults. This approach aims to enhance their contribution to societal development and prevent aging from becoming a health and social security crisis (WHO, 2020). To effectively promote healthy aging in individuals with chronic conditions, it is crucial to understand their perspectives on healthy aging as a foundation for intervention. The World Health Organization indicates that most people can expect to live beyond the age of 60, with an average of 22 additional years after reaching 60 (Seah et al., 2021; WHO, 2020). 

While everyone over the age of 65 is chronologically classified as an older person, their physical, psychological, and functional characteristics, as well as socio-economic status, can vary significantly. An individual who has maintained a health-conscious lifestyle with a balanced diet, adequate physical activity, and strong social support will face different challenges and coping levels in old age compared to someone who grew up in a disadvantaged socio-economic environment without access to adequate nutrition (Kahveci & Yeniçeri, 2023; Türkkan, 2021). 

Healthy aging has become a critical goal to prevent the widespread occurrence of multiple chronic and debilitating conditions among older adults. It is defined as “the process of developing and maintaining the functional ability that enables well-being in older age” (WHO, 2015). By adapting and compensating during the aging process, older adults can achieve healthy aging by enhancing their physical, social, and mental health to facilitate active participation in society (Wang et al., 2023). 

Numerous studies have explored older adults’ perceptions of aging and healthy aging (Bacsu et al., 2014; Guell et al., 2016; Seah et al., 2021; Waites & Onolemhemhen, 2014; Sabatini et al., 2024). These studies identify factors associated with healthy aging, such as social interaction, staying active, independence, an optimistic outlook, and cognitive health. Cultural influences on perceptions of healthy aging have also been reported (Bacsu et al., 2014; Waites & Onolemhemhen, 2014). 

There are not many good and accurate ways to test people’s thoughts on the idea of healthy aging though. Specifically, it has been found that there is no original scale made just for measuring how older persons with chronic conditions feel about healthy aging. According to the literature, most scales that measure how people think about healthy aging were designed in distinct cultural settings (Bacsu et al., 2014; Waites & Onolemhemhen, 2014). This makes it very hard to figure out how older people in Turkey think about healthy aging and to come up with good ways to help them in this area. This shows that there is a big gap in the literature when it comes to the need for a valid and reliable Turkish scale to measure how older persons with chronic diseases see healthy aging. The goal of this study is to test the Turkish version of the Healthy Aging Perception Scale for older persons with chronic conditions to see if it is valid and reliable.

## 2. Materials and Methods

### 2.1. Participants and Sampling

The study population consisted of 23,932 older adults, aged 65 and above, who applied to family health centers affiliated to Erzurum Public Health Directorate between November and December 2023. Bryman and Cramer (2005) stated that the study group should consist of at least five times the number of scale items, and Pallant (2017) suggested that the sample should include at least 150 people and at least five people for each variable (Bryman & Cramer, 2005; Pallant, 2017). It has also been suggested in different sources that the number of data required should be at least 5 times per item for exploratory factor analysis (EFA) and at least 200 samples for confirmatory factor analysis (CFA) (Bryant & Yarnold, 1995; Hair et al., 2010). Data were collected through face-to-face interviews with 424 older adults aged 65 years and older, who had at least one chronic disease, did not have cognitive problems, were registered at the designated family health center on the study dates, and agreed to participate in the study. In this study, 210 people were randomly selected for EFA and 214 for CFA (Hair et al., 2010; Koyuncu & Kılıç, 2019). 

### 2.2. Design and Instrument

The Sociodemographic Information Form and Healthy Aging Perception Scale was used to collect data for the study. The Sociodemographic Information Form is a form prepared by the researchers in accordance with the literature, which asks about socio-demographic characteristics such as age, gender, and education level. The Healthy Aging Perception Scale was developed by Wang et al. (2023). It consists of 19 items and 4 sub-dimensions. These sub-dimensions are “changes caused by physical and psychological illness”, “environmental changes caused by illness”, “changes in social relationships caused by illness”, and “changes in family life caused by illness”. A 5-point Likert scale was used to rate the statements in the scale (1 = strongly disagree, 2 = disagree, 3 = no opinion, 4 = agree, and 5 = strongly agree). The items numbered 1–5 are reverse-coded. To calculate the total score, the items are summed and then divided by the number of items. Higher scores indicate better perspectives on healthy aging. 

### 2.3. Translation and Cultural Adaptation

We obtained permission from the developer of the instrument to translate the Healthy Aging Perception Scale into Turkish. In the process of translating the scale, the translation–back-translation method proposed by Brislin and Beaton, Bombardier, Guillemin and Ferraz was used (Beaton et al., 2000; Brislin, 1986). First, two native Turkish-speaking bilingual experts with experience in English translated the English version of the scale into Turkish. Then, a third expert compared the two translations of the translated scale with the original version. Second, a native English bilingual translator independently translated it back into English. A bilingual, PhD-educated nursing researcher independently compared the back-translated scale with the original English version and did not recommend the addition or deletion of any items.

### 2.4. Psychometric Evaluation of the Scale

#### 2.4.1. Content Validity

To test whether the Healthy Aging Perception Scale is appropriate for Turkish older adults, a total of 11 experts (five professionals in public health nursing, three professionals in internal medicine nursing, and three experts in gerontology), including nursing professors and nursing teachers with experience in geriatric nursing, were invited to evaluate the Item Content Validity Index (I-CVI) and the Content Validity Index (S-CVI) of the scale. They rated the relevance of the items on a 4-point scale. The I-CVI is the ratio of the number of experts who rated each item three or four points to the total number of experts. The item was analyzed using the Item Content Validity Index (I-CVI) and the Scale Content Validity Index/Average (S-CVI), and item modification or deletion was considered if the I-CVI of the item was less than 0.80 or the S-CVI/Average was less than 0.90 (Polit & Beck, 2008). 

#### 2.4.2. Pilot Study

This was conducted with 55 older people who had registered at the Family Health Centre. None of the samples from the pilot study were included in the study. The participants were informed about the pilot study by ethical rules. As a result of the pilot study, no changes were made to the scale items as the items were understandable. 

#### 2.4.3. Construct Validity

EFA and CFA were used to test and assess the factor construct validity of the scale. Principal Component Analysis and a 25-degree varimax axis rotation was performed during EFA. Prior to examining the factor structure of the Healthy Aging Perception Scale, Kaiser–Meyer–Olkin (KMO) and Bartlett’s sphericity tests were conducted to assess the sample size and suitability of the instrument for factor analysis. The KMO index value used to determine the adequacy of the sample size was expected to be 0.70 and above. The statistical significance of Bartlett’s sphericity test indicates that the dataset is suitable for factor analysis (Seçer, 2018). The Maximum Likelihood method was used in the CFA. CFA was performed to confirm the factor structure obtained with EFA (Seçer, 2018). It has been reported in the literature that CFA fit indices should be (a) chi-square degrees of freedom (χ^2^/df) less than or equal to three; (b) root mean square error of approximation (RMSEA) less than or equal to 0.05; (c) goodness-of-fit index (GFI), adjusted goodness-of-fit index (AGFI), Tucker–Lewis index (TLI), comparative fit index (CFI), and above (Alavi et al., 2020; Rappaport et al., 2020). For convergent validity of the Healthy Aging Perception Scale, AVE and CR values of the items were calculated. 

#### 2.4.4. Reliability of the Scale

To test the scale’s reliability, internal consistency analysis (to determine item reliability and homogeneity), item–total score correlation, intraclass correlation coefficient (ICC), and test–retest were analyzed. For test–retest analysis, 65 people were randomly included from the same sample. The ICC reflects both the correlation and the degree of agreement between the measures. Hotelling’s T2 test was used to test whether the item means differed from each other (Seçer, 2018). 

### 2.5. Data Analysis

The collected data were analyzed using SPSS 27.0 and AMOS 22.0. The characteristics of the participants were analyzed using descriptive statistics such as frequency, percentage, mean, and standard deviation, and the differences between the EFA and CFA groups were analyzed using chi-squared and *t*-tests. In the validity and reliability analysis of the scale, Cronbach’s alpha coefficient, item–total score correlation, intraclass correlation coefficient and test–retest reliability methods, language and content validity, and explanatory and confirmatory factor analysis were used. 

## 3. Results

### 3.1. The General Demographics of Older Adults

The study determined that the EFA group’s mean age was 71.60 ± 6.83, the mean number of chronic diseases was 2.14 ± 0.47, 61% were female, 58.1% were primary school graduates, and 84.8% were married. It was found that 46.7% of the older adults had a low income, and 97.1% were not working. The mean age of the CFA group was 70.05 ± 7.54, the mean number of chronic diseases was 2.56 ± 1.07, 59.3% were female, 61.3% were primary school graduates and 92.2% were married. It was found that 58.8% of the older adults had income equal to expenses, and 91.2% were not working. There were no statistically significant differences between the groups (Table 1). The mean total score of the HAPQ was 3.47 (±0.54), and the mean scores of the four subscales ranged from 3.28 to 3.60 (Table 1).

### 3.2. Analysis of Psychometric Properties

#### 3.2.1. Content Validity

The scale was sent to 11 experts in the field to assess its content validity. The response rate was 90.9% (10 experts), the I-CVI ranged from 0.858 to 1.000, and the S-CVI was 0.924.

#### 3.2.2. Construct Validity

First, EFA was conducted to verify construct validity. During factor determination, eigenvalues for items were checked to be 1 or higher. The items’ loadings were required to be at least 0.30, with a minimum difference of 0.10 between factors to avoid overlapping items (Çokluk et al., 2018; Seçer, 2018). Additionally, a 25-degree varimax rotation was applied to facilitate construct validity.

The sixth item, which had a difference of more than 0.10 between the loadings of the items in the two factors, that is, the sixth item, which showed overlapping item characteristics, was removed from the scale. EFA was performed again as a result of the repeated EFA. The KMO value of the scale was 0.811, and Bartlett’s sphericity test was significant (χ^2^ = 3660.803, *p* < 0.001), indicating that the data were suitable for EFA. As a result of the EFA for the 18-item scale, a four-factor structure with an eigenvalue exceeding 1 was obtained, and it was determined that the factor loadings ranged between 0.834 and 0.637 and the total variance explained was 64.619%. The factors retained their original structure after the sixth item was removed (Table 2). 

Looking at Table 2, the first factor consisted of items 1–5. It consisted of items 1–5 and was named “Physical and psychological changes caused by the disease” as in the original. This factor explained 21.984% of the total variance. Factor 2 consisted of items 7–10, 16 and was named “Changes in the living environment due to the disease,” as in the original. This factor explained 17.610% of the total variance. Factor 3 consisted of items 13–15. It consisted of items 13–15 and was named, as in the original, “Changes in social relationships due to the illness”. This factor explained 16.631% of the total variance. Factor 4 consisted of items 11, 12, and 17–19 and was named “Changes in family life caused by the illness” as in the original. This factor explained 8.394% of the total variance.

**Figure 1:**
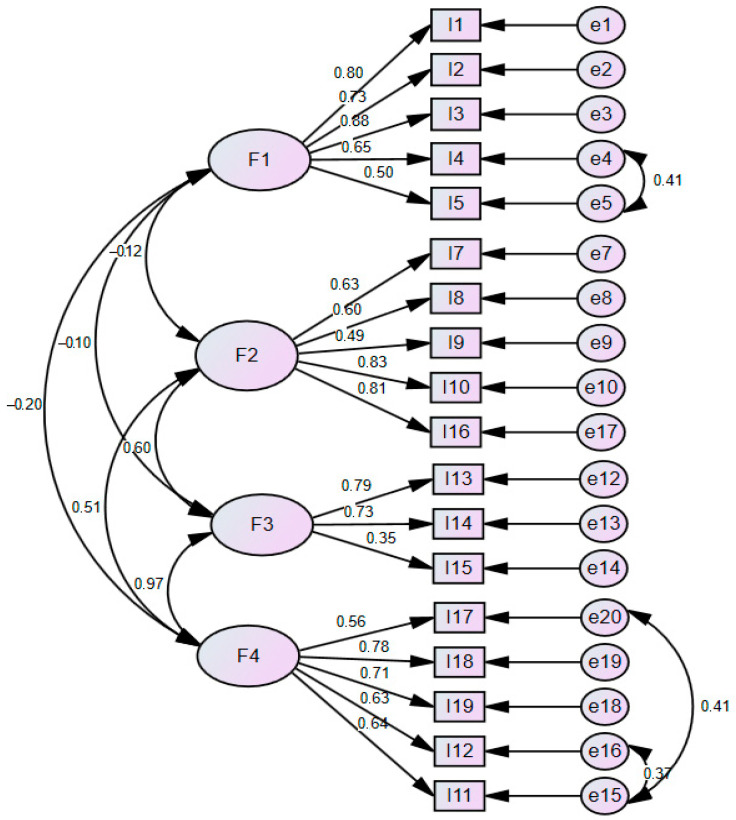
Path diagram of healthy aging perception scale.

CFA was performed on the 18-item and four-factor structure with 214 data not included in the EFA. The 18-item scale had standardization coefficients greater than 0.35. The CFA goodness of fit indices were found to be at an acceptable level. CMIN/DF = 2.834, RMSEA = 0.05, CFI = 0.929, TLI = 0.910, NFI = 0.920, and GFI = 0.901. The selected fit indices showed that the factorial model provided a good fit and confirmed the results of the EFA. The significant values and goodness of fit obtained for the fit indices are presented in Table 3. The fit values were evaluated, taking into account the Byrne reference values (Byrne, 2010). The path diagram showing the factor structure of the items is given in Figure 1. AVE values for each factor ranged from 0.43 to 0.52. CR scores ranged from 0.67 to 0.84 (Table 3). 

The correlation matrix is presented in Table 4, illustrating the relationships between factors. The factors had a moderate positive correlation (*p* < 0.001) (Table 4). 

#### 3.2.3. Internal Consistency

The internal consistency coefficient of the four-factor scale was 0.826. The internal consistency coefficients of the subscales were physical and psychological changes caused by the disease 0.908, changes in living environment caused by the disease subscale 0.860, changes in social relationships caused by the disease subscale 0.767, and changes in family life caused by the disease subscale 0.923. The values obtained show that the scale is highly reliable (Özdamar, 2017). 

The Hotelling T2 value was 468.136, *p* < 0.001. The difference between the Healthy Aging Perception Scale item means was significant. Corrected item–total correlations were between 0.395 and 0.555, indicating good internal consistency (>0.30 for all items).

#### 3.2.4. Test–Retest Reliability

In order to evaluate the stability of the Turkish form of the scale over a 2-week period, the intraclass correlation coefficient was examined in the test–retest analysis conducted with 65 randomly selected participants (r = 0.822, *p* = 0.0001).

## 4. Discussion

Healthy aging is a multidimensional concept that encompasses the prevention of disease and disability, maintaining high levels of physical and cognitive function, and active participation in social and productive activities. Turkey, with its growing older adult population, aims to develop a scale with Turkish validity and reliability to assess the perspectives of older individuals with chronic diseases on healthy aging. To achieve this, the face validity of the scale developed by Wang et al. (2023) was confirmed through expert evaluations. In this study, the Item Content Validity Index (I-CVI) ranged from 0.858 to 1.000, and the Scale Content Validity Index (S-CVI) was 0.924. In the original scale, these values ranged from 0.8 to 1.000, with the literature suggesting that a value of 0.8 and above is sufficient (Waltz et al., 2010). 

The average total score on the HAPQ in this study was 3.47 (±0.54), and the average score on each subscale was between 3.28 and 3.60. The original study by Wang et al. (2023) found a mean total score of 3.34 (Wang et al., 2023). These results show that older persons in Turkey with chronic conditions have a somewhat favorable view of healthy aging, and their overall view is a little better than the original sample’s. The fact that the subscale means are so close together in our study shows that participants think that changes in family life, social interactions, living conditions, and physical and mental health are all equally essential. This balanced profile suggests that efforts to encourage healthy aging should be comprehensive and include all key areas instead than just one. You can use the HAPQ in both clinical and community settings to find out how different people or groups (such as those with lower incomes, those who live alone, or those who do not have a lot of social support) see things.

The EFA and CFA results showed that the Turkish version of the scale was in agreement with the model developed by Wang et al. (2023). Item 6 from the original 19-item scale with four sub-dimensions was taken out because it had a low factor loading. The question, “Will your mood change when the doctor tells you about some changes in your conditions at your next visit?” may not have made sense to Turkish participants because of cultural differences in how people talk to doctors and deal with disease. It is likely that people in Turkey did not understand this issue clearly or did not think it was important. The fit indices for the new 18-item scale were acceptable after this item was taken out (Çokluk et al., 2018).

Subsequent reliability analyses revealed that the internal consistency coefficient for the entire scale was 0.826. The Cronbach’s alpha coefficients for the scale’s reliability ranged from 0.75 to 0.92, which Alpar (2018) considers highly reliable (Alpar, 2018). In the original study of the scale, it was reported that the Cronbach alpha coefficient varied between 0.71 and 0.89 (Wang et al., 2023).

Additionally, the Composite Reliability (CR) and Average Variance Extracted (AVE) coefficients for the three sub-dimensions exceeded 0.70, indicating acceptable reliability levels. The condition CR ≥ AVE ≥ 0.05 must be met for convergent validity. However, if AVE values are below 0.5, a CR ≥ 0.7 is acceptable for convergent validity (Fornell & Larcker, 1981; Hair et al., 2010). 

Furthermore, the test–retest analysis yielded an Intraclass Correlation Coefficient (ICC) of 0.822, which is considered sufficient, as values of 0.80 and above are deemed acceptable in the literature (Koo & Li, 2016). 

### Limitations

This study presents both limitations and strengths. A primary limitation is that the sample is drawn from a single region and population, resulting in a homogeneous group that restricts the generalizability of the findings. Future research should aim to explore the perceptions of healthy living among older adults with chronic diseases using a larger and more diverse sample. On the strength side, the study successfully determined the test–retest reliability of the Healthy Aging Perception Scale, which is a crucial aspect of measurement reliability. Additionally, the study’s use of various psychometric validation methods, including convergent and factorial validity, enhances its robustness and credibility. Another limitation of this study is that we did not assess criterion validity; for example, we did not examine how HAPS scores correlate with external health indicators or other well-being measures. While this is not required for a psychometric validation study, future research could address this by evaluating the relationship between HAPS scores and relevant health outcomes or established scales.

## 5. Conclusions

The Healthy Aging Perception Scale has been validated as a reliable and valid tool for assessing the perceptions of healthy aging among older individuals with chronic diseases. Understanding these perceptions is crucial for effectively promoting healthy and independent living in this demographic. As the first instrument specifically designed to measure older adult perceptions of healthy aging in Turkey, the Healthy Aging Perception Scale fills an important gap in Turkish gerontology by providing a culturally appropriate, validated assessment tool. However, further validation in broader and more diverse populations is recommended before widespread use. This will ensure the scale’s applicability and robustness across different settings and subgroups.

## Figures and Tables

**Table 1:** Frequency distribution of demographics characteristics.

Characteristics	Participants in EFA (n = 210)		Participants in CFA (n = 214)		t/χ^2^ (*p*)
**Average Age**	71.60 ± 6.83		70.05 ± 7.54		1.148 (0.285)
**Number of chronic diseases**	2.14 ± 0.47		2.56 ± 1.07		0.473 (0.563)
	**n**	**%**	**n**	**%**	
**Gender**					
Men	82	39.0	83	40.7	0.116 (0.764)
Women	128	61.0	121	59.3	
**Education**					
Illiterate	64	30.5	48	23.5	3.127 (0.209)
Primary education	122	58.1	125	61.3	
High school and above	24	11.4	31	15.2	
**Married**					
Married	178	84.8	188	92.2	1.521 (0.910)
Single	32	15.2	16	7.8	
**Income**					1.205 (0.720)
Less	98	46.7	71	34.8	
Middle	96	45.7	120	58.8	
Very much	16	7.6	13	6.4	
**Employment Status**					1.745 (0.900)
Yes	6	2.9	18	8.8	
No	204	97.1	186	91.2	
					**X ± SS**
Healthy aging perspectives questionnaire (HAPQ)					3.47 ± 0.54
Physical and psychological changes caused by disease					3.55 ± 1.03
Living environment changes caused by the disease					3.60 ± 0.79
Social relationship changes caused by the disease					3.43 ± 0.85
Changes in family life caused by the disease					3.28 ± 0.89

**Table 2:** Healthy Aging Perception Scale Rotated Factor Pattern Matrix (n = 210).

Items	F 1	F 2	F 3	F 4
I1—Do you think your physical strength is getting worse after having the chronic disease?	0.834			
I2—Do you think you cannot do whatever you want as you used to after having the chronic disease?	0.821			
I3—Do you think the disease has brought you some trouble in your daily life?	0.870			
I4—Do you think the long-term and regular medication for the disease has brought you some trouble?	0.745			
I5—Do you think that even with regular medication, it is still impossible to control the changes in the disease?	0.572			
I7—Do you accept the doctor’s instruction and change your lifestyle for the disease?		0.566		
I8—Would you change your lifestyle and start regular exercise or proper diets for the disease?		0.737		
I9—Would you use appropriate aids for the disease, such as crutches, walkers or other aids to help yourself?		0.645		
I10—Will you learn new things depending on your conditions?		0.809		
I16—Did your family help improve the environment and space to make your life easier because of the disease?		0.707		
I13—Will changes in your relatives’ or friends’ diseases influence your thoughts on life?			0.743	
I14—Do you feel needed from the process of serving the society or your family members?			0.654	
I15—Do you feel satisfied from the process of serving the society or your family members?			0.649	
I11—Do you think that you spent too much time on work when you were young and now at an older age, you should spare more time to arrange your life?				0.787
I12—Do you think that there is no need to hold on to unhappy things at an older age?				0.770
I17—Do you feel happier when staying out of your child/children’s business?				0.722
I18—Do you think that when you are older, you will learn to shift the focus of your life from the child/children to yourself?				0.752
I19—Do you think husband and wife should arrange common activities?				0.637
Eigenvalue	5.532	3.050	1.839	1.211
Explained variance (%)	21.984	17.610	16.631	8.394
Total explained variance (%)		64.619		

**Table 3:** Results of confirmatory factor analysis.

Factors	Items	Estimate	S.E.	Critical Ratio	CR	AVE
1	I1	0.800		9.46	0.84	0.52
	I2	0.727	0.053	9.43		
	I3	0.885	0.055	9.59		
	I4	0.654	0.058	10.12		
	I5	0.496	0.062	8.47		
2	I7	0.626		9.91	0.80	0.47
	I8	0.596	0.083	8.85		
	I9	0.485	0.086	9.51		
	I10	0.826	0.089	9.88		
	I16	0.791		10.22		
3	I13	0.729	0.064	10.27	0.67	0.43
	I14	0.349	0.063	10.20		
	I15	0.636		10.07		
4	I11	0.632	0.07	13.12	0.80	0.45
	I12	0.811	0.09	9.78		
	I17	0.706	0.095	10.28		
	I18	0.777	0.097	13.12		
	I19	0.558	0.083	10.53		
Fitness index	CMIN/DF	RMSEA	CFI	TLI	NFI	GFI
Reference value	<3.0	<0.08	>0.90	>0.90	>0.90	>0.90
Model	2.834	0.05	0.929	0.91	0.89	0.90

**Table 4:** The squares of correlation coefficients between factors.

	Factor 1	Factor 2	Factor 3	Factor 4
Factor 1	1			
Factor 2	0.314 *	1		
Factor 3	0.360 **	0.583 **	1	
Factor 4	0.346 **	0.437 **	0.712 **	1

* *p* < 0.05. ** *p* < 0.001.

## Data Availability

The raw data supporting the conclusions of this article will be made available by the authors on request.

## Abbreviations

The following abbreviations are used in this manuscript:

EFA	Exploratory factor analysis
CFA	Confirmatory factor analysis
ICC	Intraclass correlation coefficient
RMSEA	Root mean square error of approximation
GFI	Goodness-of-fit index
AGFI	Adjusted goodness-of-fit index
TLI	Tucker–Lewis index
CFI	Comparative fit index
IFI	Incremental fit index
PGFI	parsimonious goodness-of-fit index
PNFI	parsimonious normed-of-fit index
